# Characteristics of online shopping behaviour among Tunisians consumers

**DOI:** 10.1192/j.eurpsy.2024.1194

**Published:** 2024-08-27

**Authors:** M. Mnif, F. Guermazi, R. Masmoudi, W. Abid, I. Feki, I. Baati, J. Masmoudi

**Affiliations:** CHU Hedi Chakeur psychiatry A department, Sfax, Tunisia

## Abstract

**Introduction:**

During the COVID-19 pandemic confinement, the number of people shopping online has increased all over the word. To date, little is known about the online shopping behaviours of Tunisians consumers.

**Objectives:**

Evaluate the characteristics of internet shopping among Tunisian consumers.

**Methods:**

A cross-sectional, descriptive and analytical study was conducted among subjects who had already made at least one online purchase. Data was collected using a self-questionnaire published by GOOGLE FORMS. We used a survey form collecting socio-demographic data, personal history and characteristics of online shopping behaviour.

**Results:**

A total of 137 participants aged 34.62 ± 9.82 years took part in this study.

All participants had made at least one online purchase, with 43.8% (N=60) purchasing “More than once a year”. The products purchased were most often textiles and shoes (50.4%; N=69). The main reasons consumers gave for buying online were special offers (37.2%, N=51), reduced prices (25.5%, N=35) and free delivery (14.6%, N=20). Almost half of the participants (N=63; 46%) said that they had visited physical shops less since they started shopping online. Regarding the average online shopping budget, 44.5% of consumers (N=61) spent less than 50 dinars/month and 18.2% (N=16) did not use all the products they bought online. Almost half of participants (N=68, 49.6%) feared that their credit card information would be at risk. The majority of respondents (88.9%) thought they might receive a faulty product following online shopping.

**Conclusions:**

Our study has enabled us to identify certain factors that may act as a blocker for online purchasing. So that, stablishing strategic actions for the continuous improvement of online shopping services with the reduction of subjectivity in customer perception will be helpful.

**Disclosure of Interest:**

None Declared

